# Study of vacuolating cytotoxin A (vacA) genotypes of ulcerogenic and non-ulcerogenic strains of *Helicobacter pylori* and its association with gastric disease

**DOI:** 10.1016/j.sjbs.2023.103867

**Published:** 2023-11-04

**Authors:** Mushtak T.S. Al-Ouqaili, Rawaa A. Hussein, Yasin H. Majeed, Farah Al-Marzooq

**Affiliations:** a Department of Microbiology, College of Medicine, University of Anbar, Al-Anbar Governorate, Ramadi, Iraq; b Department of Clinical Laboratory Sciences, College of Pharmacy, University of Anbar, Al-Anbar Governorate, Ramadi, Iraq; c Department of Internal Medicine, College of Medicine, University of Anbar, Al-Anbar Governorate, Ramadi, Iraq; d Department of Microbiology and Immunology, College of Medicine and Health Sciences, United Arab Emirates University, Al-Ain, United Arab Emirates

**Keywords:** *Helicobacter pylori*, *vacA*, PCR, Clarithromycin, Peptic ulcer

## Abstract

Globally, Helicobacter pylori (*H. pylori*), a stomach pathogen, is present in around 50 % of the population. This bacterial infection produces persistent inflammation, which significantly raises the risk of duodenal, gastric ulcer, and stomach cancer. The goal of this study is to identify the vacA genotypes in *H. pylori* and analyze how they relate to medical conditions brought on by the bacteria and clarithromycin resistance. PCR was used to describe 115 endoscopic stomach samples from infected patients and identify vacA gene. Of the 115 research participants, *H. pylori* was found in 81 (70.4 %) of them. Of the isolated cultures, only 38 (69.1 %) were resistant to clarithromycin. VacA was discovered in 55 (67.9 %) of the samples that had *H. pylori* in them. Patients with gastritis were more likely to have s2m2 strains of infection (66.7 %), while those with gastric and duodenal ulcers were more likely to have s1m1 strains (64.7 %). VacA-positive *H. pylori* strains (60 % n = 33) were more resistant to clarithromycin versus (19.2 % n = 5) for *vacA-*negative bacteria. Clarithromycin resistance was significantly linked to *vacA s2m2* in *H. pylori* isolates (75.9 %). According to the study's results, the vacA variants *s1m1* and *s2m2* have a strong connection with the emergence of *H. pylori* infections that cause peptic ulcer disease in the population of Iraq. Genetic testing is essential in predicting both the course of treatment and the outcome of *H. pylori* disease.

## 1 Introduction

The disorders *Helicobacter pylori* illness, ulceration of the stomach, and prolonged gastritis are all related. As a result, the WHO has categorized it as a class I carcinogen since 1994. (Blanchard and Czinn, 2017). Surprisingly, main symptoms of the condition occur far less frequently in some parts of the world than in others, despite the fact that this bacterial infection is relatively common in some countries. Variations in the signs and symptoms of *H. pylori* infection may be caused by the virulence factors of different bacterial strains, in addition to the characteristics of the host and dietary habits. (Hassan khater and AlFaki, 2022). Many of the virulence characteristics of *H. pylori* allow the bacteria to stick to gastric epithelial cells, persist in the acidic environment of the stomach, and damage affected tissues (Baj et al., 2021). One of these virulence factors is vacA, a pore-forming toxin that results in the formation of vesicles in the cells of the gastric epithelium. The vacA gene produces a protein that may be involved in a number of cellular processes, such as the suppression of T-cell activation and multiplication as well as the start of a pro-inflammatory reaction (Akeel et al., 2019). Nearly all isolates of *H. pylori* express this pore-forming gene, however there are considerable differences across strains in terms of their capacity to cause cell vacuolization (Akeel et al., 2019). The *vacA* gene, which has a wide range of polymorphic rearrangements possible, has a genomic architecture that is varied, which contributes to this variance (Soyfoo et al., 2021). The signal region (*s*) and middle (*m*) regions of this toxin were discovered in early research in the field. Either the *s1* or *s2* kind is encoded in the s-region. The m-region encodes the m1 or m2 genes. The *vacA* type *s1* isolates are more frequently seen in peptic ulcer disease and seem to be more virulent than the *s2* isolates. More epithelial stomach injury is brought on by vacA *m1* strains than vacA *m2* strains The interaction of the s- and m-region genetic types, which is connected to the virulence of the *H. pylori* strain, regulates the production of cytotoxins. In contrast to the *s1m2* strains, which either generate a moderate quantity or very little toxin, and the *s2m2* isolates, which either create nearly no toxin or very little toxin, the s1m1 isolates produce huge amounts of toxin. (El-Shenawy *et al.*, 2017a). Since it is one of the main factors in unsuccessful therapies, antibiotic resistance in *H. pylori* raises serious concerns. *H. pylori* resistance is significantly more common in developing countries than it is in wealthy ones (Savoldi et al., 2018). Resistance to medications in *H. pylori* is typically acquired via chromosomal changes, through vertically transmitted DNA point mutations, as opposed to plasmid acquisition. (Zhang et al., 2020). The relation between bacterial virulence and resistance to drugs is critical for predicting treatment response and the course of an illness brought on by *H. pylori* (Vital et al., 2022).

Our investigation aimed to identify vacA genotype combinations in *H. pylori* isolates from ulcerogenic and non-ulcerogenic strains that were resistant to clarithromycin, as well as their relationships to various genotypic markers and gastroduodenal diseases.

## 2 Materials and procedures

### 2.1 Patients and clinical samples

Those individuals who signed up for this research project between January 2020 and February 2021 are referred to as the cohort. A total of 115 patients visited the Ramadi Teaching Hospital (https://maps.app.goo.gl/UpCGFn6vua3hkotg7) in Iraq gastrointestinal endoscopy were recruited. Patients' ages ranged from 17 to 69, and 80 (69.6 %) of them were men and 35 (30.4 %) were women. PPI, H2 antagonists, antibiotic therapy, NSAIDs medications were all viewed as exclusion criteria. People with kidney failure, cirrhosis, and pregnancies in the month before endoscopy were also excluded from the trial (Husseinet al., 2022a). Stomach tissue samples were obtained from the stomach corpus and antrum during a routine endoscopy by a qualified medical professional (a gastroenterologist), who then placed them for transport in sanitary tubes containing BHI medium with 5 % fetal bovine serum. Clinical assessment and assessment of the endoscopic and histological abnormalities was also done to determine the diagnosis in each patient (Hussein et al., 2021a, Hussein et al., 2021b).

### 2.2 Ethical approval

All patient-involved study methods were accepted by the University of Anbar's Ethical Approval Committee on November 20, 2019, in Ramadi, Iraq. All subjects in the study gave proper, signed informed consent.

### 2.3 Identification of *H. pylori* bacteria that are resistant to clarithromycin

In a previous study by our team, *H. pylori* could be identified in biopsies *(*Hussein et al., 2021a, Hussein et al., 2021b). Determination of antibiotic susceptibility was done using the Epsilometer test to determine the (MIC). Clarithromycin resistance in *H. pylori* has been linked to certain point mutations in the 23srRNA, particularly A2143G and A2144G. This was discovered in accordance with our earlier research on the same strains (Hussein et al., 2021a, Hussein et al., 2021b).

### 2.4 Detection of ulcerogenic and non-ulcerogenic strains of *H. pylori*

Genomic DNA was extracted using the SaMag-12 automatic nucleic acid extraction device and SaMag Tissue DNA extraction kits (Sacace, Italy) (Samaga, Cepheid, Italy) (Al-Kubaisy et al., 2020). At a temperature of −20 °C, the extracted DNA was kept. To assess the quality of the sample for future applications, the concentration of extracted DNA was determined using a Quantus TM Fluorometer (Promega, USA) (Khalaf and Al-Ouqaili, 2018). The polymerase chain reaction (PCR) was conducted with the *vacA* and *vacA* allele primers (Alpha DNA Co., Canada) as specified in Table 1.

**Table 1:** The primers utilized in the investigation.

**Gene target**	**Primer Sequence (5′-3′)**	**PCR product size (bp)**
***VacA***	F: GAG CGA GCT ATG GTT ATG AC	229
	R: ACT CCA GCA TTC ATA TAG A	
***VacA genotype*** **)*s1*, *s2*)**	F: ATGGAAATACAACAAACACAC	*s1:* 259/*s2:* 286
	R: CTGCTTGAATGCGCCAAAC	
***VacA genotype*** **(*m1*, *m2*)**	F: CAATCTGTCCAATCAAGCGAG	*m1:* 290/*m2:* 350
	R: TGAGTTGTTTGATATTGAC	

AccuPower® PCR PreMix was used in the PCR process to identify the vacA gene and (s1, s2, m1, m2) with a final volume of 20 μl, including 2 μl of forward and reverse primers, 5 μl of genomic DNA as a template, and 11 μl molecular biology water. To amplify the *vacA* gene, the Polymerase Chain Reaction thermal cycler (Esco, USA) was programmed for amplification including 35 cycles of denaturation (30 s at 95 °C), annealing (30 s at 53 °C), extension (4 min at 72 °C), and final extension (5 min at 72 °C) (Chisholm et al., 2001). While the thermal cycler (Esco, USA) was programmed to perform 35 denaturation cycles according to (Atherton, 1997).

### 2.5 Analytical statistics

All of the data were examined using the SPSS program. Diagnoses, RT-PCR findings, patient age group and gender, as well as their frequency of occurrence, were displayed. Frequencies and percentages were shown as tables and figures for case descriptions. Through the use of cross-tabulation analysis, Chi-Square analysis was utilized to examine associations between the category variables indicated above. Standard deviation and mean ages are compared. Descriptive statistics were used to determine patient deviations based on diseases.

## 3 Result

### 3.1 Patients’ characteristics

Out of 80 males, 60 (52.17 %) had *H. pylori* infection and 20 (17.39 %) did not have, while in females, *H. pylori* positive was observed in 21 (18.26 %) and negative in 14 (12.17 %).

Our earlier studies revealed that 81 (70.4 %) of the 115 participants in the current research had *H. pylori* (Hussein et al., 2021a, Hussein et al., 2021b) Fig. 1. A total of 60 (74.1 %) and 21 (25.9 %) specimens from males and females, respectively, were isolated and diagnosed. Males and females were 18–69 years old and 17–66 years old, respectively. Antral gastritis was diagnosed in 47 (58 %) of the 81*H. pylori* infected patients, whereas 18 (22.2 %) had combined gastric and duodenal ulcers, seven (8.7 %) had duodenitis, four (4.9 %) were normal, two (2.5 %) had combined gastritis and duodenitis, two (2.5 %) had hiatus hernia, and one (1.2 %) had esophagitis. The *H. pylori vacA* gene was detected directly using DNA isolated from gastric biopsy samples.

**Fig. 1:**
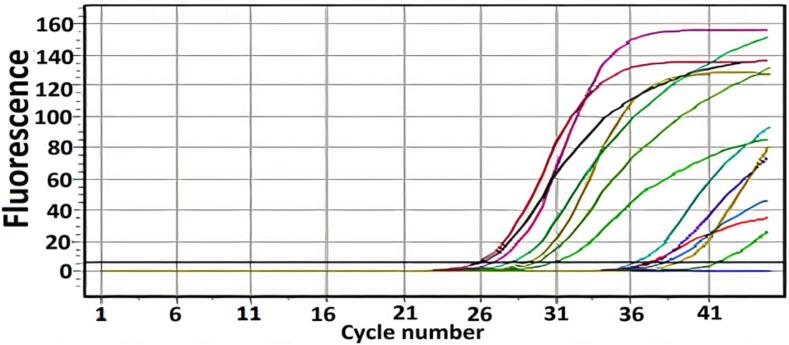
RT-PCR positive results for *H. Pylori* (*16S rRNA*).

### 3.2 The vacuolating cytotoxin *A* (*VacA*) gene

**Fig. 2:**
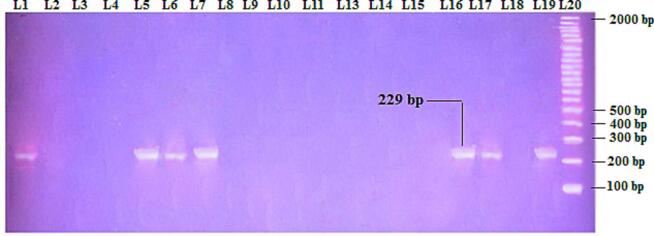
The positive result of 2 % agarose gel electrophoresis of *vacA* gene (229 bp) as appeared in lanes (1, 5, 6, 7, 16, 17 and 19) which represents study isolates (1, 4, 5, 6, 7, 10 and 11), respectively. Lane 20: represents DNA ladder as molecular weight marker (100 bp).

**Fig. 3:**
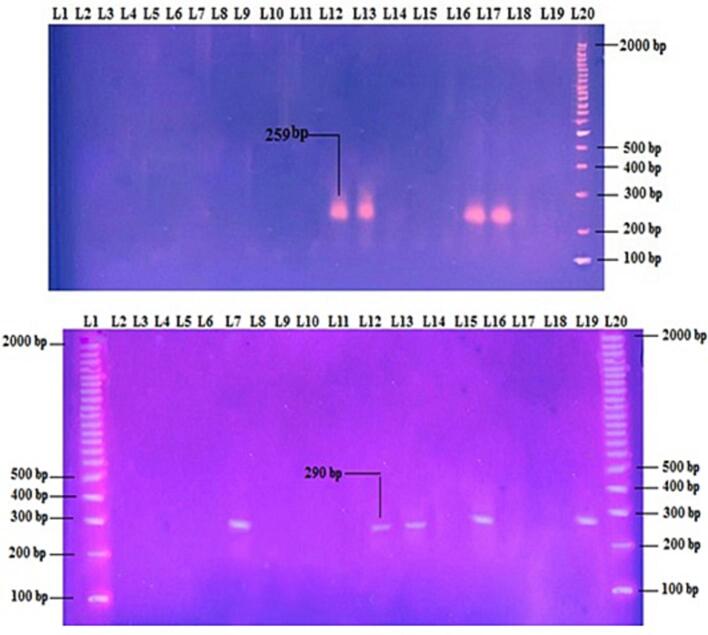
The positive results of 2 %agarose gel electrophoresis of *s1* gene (259 bp) and *m1* (290 bp) as appeared in lanes (12, 13, 17 and 18) and (7, 12, 13, 15 and 19) which represents study isolates (1, 3, 7 and 11) and (1, 3, 7, 11 and 18), respectively. Lane 20: represents DNA ladder as molecular weight marker (100 bp).

**Fig. 4:**
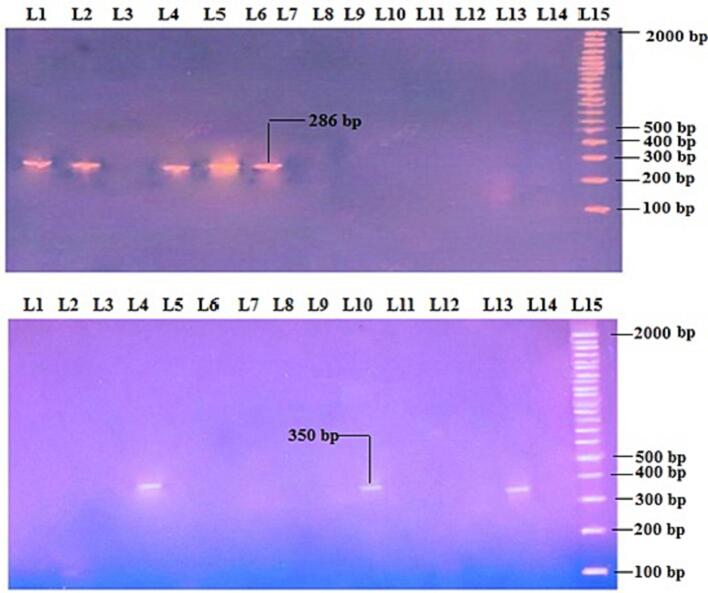
The positive results of 2 % agarose gel electrophoresis of *s2* gene (286 bp) and *m2* (350 bp) as appeared in lanes (1, 2, 4, 5 and 6) and (4, 10 and 13) which represents study isolates (6, 10, 18, 24 and 25) and (6, 10 and 24), respectively. Lane 15: represents DNA ladder as molecular weight marker (100 bp).

Of the 81*H. pylori* confirmed specimens, the vacA gene was discovered in 55 (67.9 %),(Fig. 2). The signal and middle regions of the vacA gene have been identified in each of the 55 vacA-positive specimens. Four vacA alleles were found, with the *s2m2* allele combination being the most prevalent, in 29/55 (52.7 %) potential, being the most prevalent, followed by *s1m1* 20/55 (36.4 %), *s1*m2 5/55 (9.1 %), and the *s2m1* genotype found in only one strain (Fig. 3, Fig. 4).

A total of 33/47 (70.2 %) patients with gastritis and 17/18 (94.4 %) patients with mixed gastric and duodenal ulcers tested positively for the vacA genotype. VacA *s1*, *m1*, and *m2* alleles were more prevalent among individuals with combined stomach and duodenal ulcers (at 70.6 %, 64.7 %, and 35.3 %, respectively), whereas vacA *m2* (75.8 %), *s2* (66.7 %), and *s1* (33.3 %) alleles were more prevalent in individuals with gastritis. The kind of gastrointestinal disorder and *H. pylori* infection in persons were found to be strongly correlated by two-by-two cross tabulation analysis. VacA *s2m2* and *s1m1* were frequently found in infected people who had gastritis and combined ulcers of the stomach and duodenum, respectively, in 66.7 % and 64.7 % of cases, with OR values of 14.00 (1.7787–60.2333; p 0.001) and 24 (4.7397–140.4567; p 0.001).

### 3.3 Relationship between patients’ age and *H. pylori* genotypes

In the 81 individuals who had *H. pylori* strains that were investigated, the age range of the individuals being studied was 17 to 69. Infection with this bacteria decreases with age and is more prevalent in individuals aged ≤ 37 years (56/81, 69.1 %); on the other hand, 15/81 (18.5 %) were 38–53 years old, and 10/81 (12.4 %) were ≥ 54 years. In individuals aged ≤ 37 years, the *s2* and *s2m2* genotypes were found significantly more (p < 0.0001), The genotypes *s1* or *s1m1* did not significantly correlate with this age group, as shown in Table 2.

**Table 2:** Relationship between age of patient and *H. pylori vacA* genotypes in 81*H. pylori*-positive patients.

**Age group in years**	**Number of patients with positive *Helicobacter pylori vacA* gene**							
	***s2m2***	***s2m1***	***s1m2***	***s1m1***	***m2***	***m1***	***s2***	***s1***
**≤ 37 (n = 56)**	25**	13	26	12	2	11*	24**	1
**38**–**53 (n = 15)**	4	3	4	3	–	3	4	–
**≥ 54 (n = 10)**	1	9**	4	6	3	6**	1	–

* p = 0.086;

** p < 0.0001.

### 3.4 Association between the distribution of vacA mosaicisms and clarithromycin resistance

All isolated strains of this bacteria were examined for clarithromycin resistance, which was identified in 38 (69.1 %) bacteria, while the remaining 17 (30.9 %) isolated bacteria were susceptible to the antibiotic. In total, 115 biopsies yielded 55 (47.8 %) *H. pylori* cultures. Out of 55*H. pylori* positive cultures, *vacA* gene was found in 40 (72.7 %) isolates, while another 15 (27.3 %) samples tested negative for *vacA* gene from the DNA isolated form the biopsies while being culture positive; thus, were tested for clarithromycin resistance.

**Fig. 5:**
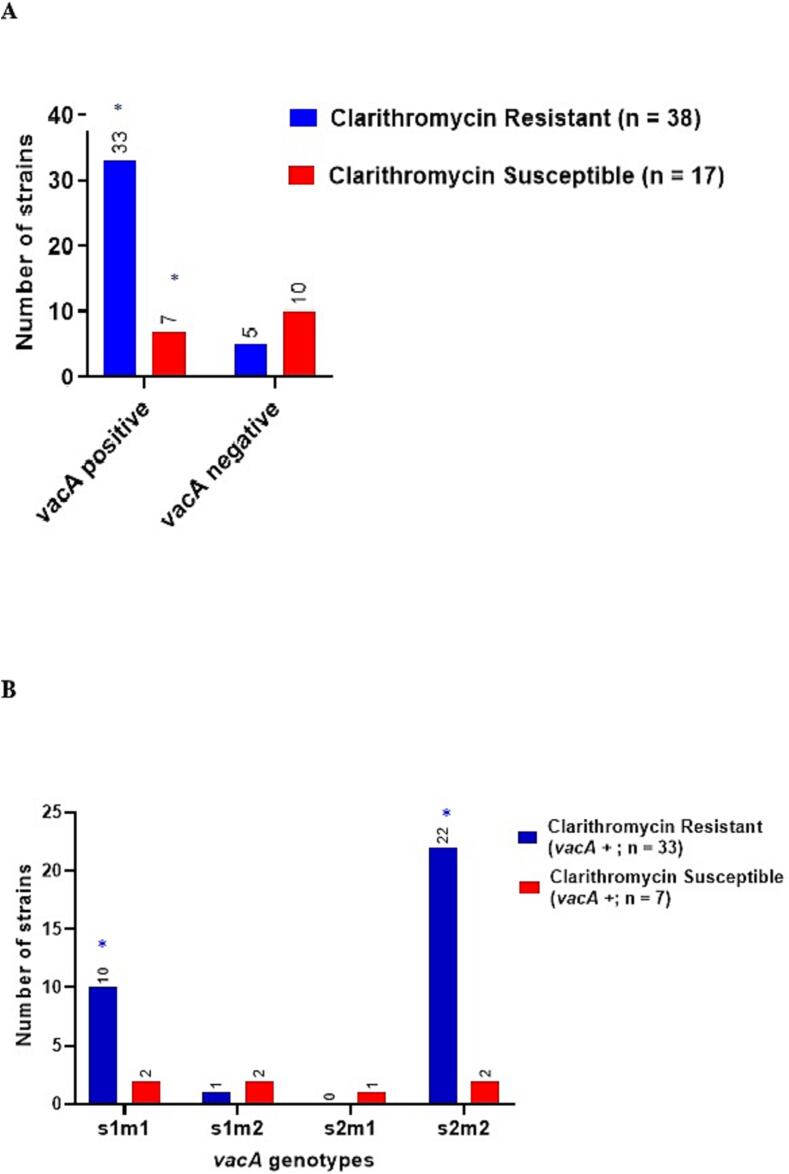
Relation between *vacA* genotype and clarithromycin resistance. A. association between *vacA* status and clarithromycin resistance in 55*H. pylori* strains. B. Association between clarithromycin resistance and *vacA* genotypes combinations in 33 *vacA* positive strains. **: p = 0.021; ***: p < 0.0001.

As shown in Fig. 4(A), *vacA* genotype was linked to clarithromycin resistance among 38 clarithromycin resistant strains. Clarithromycin resistance was found in 33 (60 %) bacteria isolates which were positive for *vacA* gene. On the other hand, only 5 (19.2 %) isolates resistant to clarithromycin tested negative for *vacA* gene. Out of the 17 strains that were susceptible to clarithromycin, only 7 were positive for *vacA* gene, while 10 were *vacA* negative. According to two-by-two cross tabular analysis, *s1* had significant association with clarithromycin resistance (OR: 5.500 (1.1453–26131, p = 0.02). Additionally, *s2* had significant association with clarithromycin resistance (OR:14.6664(2.9176–73.7288), p = 0.0003), while *m1* had significant relationship with clarithromycin resistance (OR:6.667 (1.2444–357147), p = 0.02) and *m2* had significant association with resistance (OR:11.5.00 (2.5406–52.0541), p = 0.0006) Clarithromycin resistance was consequently noticeably higher in *H. pylori* isolates that were vacA positive (p < 0.0001). Furthermore, as expected, clarithromycin resistance was significantly linked to *vacA s2m2* and *s1m1* in *H. pylori* isolates in 75.9 % and 50 % of the patients, respectively (OR: 10.00 (1.5576–642001), p = 0.0009 and (OR:22.00 (3.6286–133.3844), p < 0.0001 respectively), as shown in in Fig. 5(B).

## 4 Discussion

One of the most common infectious agents in the world is *H. pylori*, which can cause clinical disorders such chronic gastritis, which has been related to peptic ulcer disease and stomach cancer (Kishk et al., 2021). All suggestions state that elimination of this bacteria is essential for preventing carcinoma of the stomach (Mladenova, 2021). In the current study, two biopsy samples from the antral and corpus of each patient with gastrointestinal disease were collected, and positive results were corroborated by utilizing a qRT-PCR technique to find the 16S rRNA gene. Hussein et al., 2021a, Hussein et al., 2021b reported that this bacterial infection was found in 70.4 % of the patients investigated (Hussein et al., 2022a, Hussein et al., 2022b). The current finding agreed with studies that found infections caused by this bacteria in 60.9 %, 62 %, and 70 % of the subjects, respectively (El Sayed Zaki and Elewa, 2016, Abu-Zekryet al., 2013). Our result, on the other hand, was higher than that of previous Iraqi investigations by Alatbee in 2019, who found *H. pylori* infection in 51 % and 58 % (Alatbee, 2019). Socioeconomic status, geography or living conditions, and population location may all have an impact on the variation in *H. pylori* infection incidence between countries and even between populations within the same country. Infection rates in undeveloped countries are likely to be significantly higher (20–90 %) than in developed countries (10–60 %) (Alatbee, 2019). It appears that socioeconomic status, which has an impact on a population's lifestyle, can be linked to this bacterial illness. The severity of the strains, the genetic makeup of the host, and the environment all have an impact on how this bacterium's clinical symptoms develop. (Keikha et al., 2020). Gastritis was the most frequent finding on upper gastrointestinal endoscopy in *H. pylori*-infected individuals (58 %), and mixed gastric and duodenal ulcers were the second-most common finding (22.2 %). Our findings are close to those of Hassan et al. and Oraijah et al (Hassan Khater and AlFaki, 2022, Oraijahet al., 2022).

The relationship between the virulence of the *H. pylori* infection and the severity of the sickness was assessed using the vacuolating cytotoxin. The use of a genotype marker is common. Numerous epidemiological studies have discovered regional variations in its virulence factors, including mosaic combinations of the *vacA* gene alleles (Agudo et al., 2010). The examination of the vacuolating cytotoxin A mosaicism identified every feasible combination; the *s2m1* mosaicism is uncommon but has been previously described (Matsunari et al., 2016).

There may be differences in geographic origin that account for the discrepancy between the results of this study and those of several findings. The vacuolating cytotoxin, a gene in 67.9 % of the *H. pylori* strains examined, was discovered. Our findings were close to those of Al-Sabary et al., (Al-Sabary et al., 2017) from Iraq, who found *vacA* gene in 68.5 %. The virulence of *H. pylori* strains is influenced by the different combinations of the vacA s and m regions.. *In vitro,* isolates with *s1m1* genotype generate more cytotoxin than isolates with *s1m2* genotype, whereas less virulent type with *s2m2* isolates are not able to produce any cytotoxin at all. The isolated strains' vacA profiles must be determined, and these genetic combinations must be examined in light of the patients' clinical diagnoses. In their investigation, Duodenal ulcers and stomach cancer are linked to vacA s1-positive strains, according to research by Erzi and colleagues (Erzin et al., 2006). While several research from Eastern and Middle Eastern nations have revealed a link between the vacA m area and clinical outcomes, it is currently uncertain whether this association exists across all populations. Salehi and colleagues discovered that the vacA m1 and m2 frequencies were 40 % and 60 %, respectively, in a group of Iranians (Wanget al., 1998, Salehiet al., 2009). Additionally, they discovered a link between gastroduodenal disease and the vacA *m1* genotype. The *vacA m2* genotype was found to be the most common, accounting for 65.5 % of cases. Our results, which are similar to those of Akeel and colleagues, showed a substantial relationship between vacA genotypes and clinical outcomes for antral gastritis, mixed stomach and duodenal ulcers, and hiatus hernia (Akeelet al., 2019, Marie, 2012). According to our findings, most people with combined gastric and duodenal ulcers had the vacA + genotype (17/18), particularly the vacA s1m1 genotype (11/17), which was consistent with a previous study (Salehi et al., 2009), while most people with antral gastritis had the vacA *s2m2* genotype (22/33), which was similar to reports by El-Shenawy et al. Akeel et al (Akeelet al., 2019, El-Shenawyet al., 2017). Because *H. pylori* exhibits wide geographic diversity, specific genotypes may be linked to serious clinical implications in some regions of the world while appearing as less hazardous or even harmless variants in others. The reported changes in *H. pylori* virulence genes between research may result from the limits of PCR-based techniques or from variances in the experimental settings (Kishk et al., 2021).

The diversity of the vacA gene has previously been associated with regions like the Middle East, where the genotypes of the bulk of the southern and northern populations differed significantly (El-Shenawy et al., 2017). Frequencies of 45.9 % and 29.7 % for the vacA s1 and m1 genotypes were found by researchers, which is comparable to our study' detection rate for the vacA *s1* and *m1* alleles. We did not agree with El-Shenawy and colleagues findings, which showed that people in the southern Middle East who lived close to African Arabs were more likely to have cultural ties to African nations than people in the northern Middle East. The current result is also inconsistent with those reported by Erdoğdu, who found frequencies of 72.9 % and 33.6 % for the *vacA s1* and *m1* genes in Turkish patients (Erdoğdu et al., 2014). In contrast to Sallas and colleagues' and Mendoza-Cant's et al.'s outcomes, we found low s1m1 rates in the *H. pylori* + strains (Sallaset al., 2017, Mendoza-Cantú et al., 2017). Low vacA s1 m1 in the present investigation may be caused by variations in sample numbers, geographic regions, and population. Although *H. pylori* infection levels were high in the Middle East, weakly cytotoxic strains were abundant there, which could be the reason for the region's low stomach cancer incidence (El-Shenawy et al., 2017). According to our findings, the vacA s2m2 genotype had a greater detection rate than the vacA s1m1 genotype, which is regarded to be more active and capable of more severe cell damage (Sallaset al., 2017, Alduhaidhawiet al., 2022). Additionally, we found that the *s1m2* was consistent with the results of (Chisholm et al., 2001) that the least common *s2m1* in this study was comparable to Falsafi and associates and El-Shenawy and colleagues (El-Shenawyet al., 2017, Falsafiet al., 2015). The *s2* and *m2* isolates are less virulent as demonstrated by the increased risk of peptic ulcers or gastric carcinoma in those infected with the vacA s1 or m1 *H. pylori* isolates than those infected with the *s2* or *m2* strains. Studies carried out in China, Middle Eastern countries, Africa, and Western nations have revealed this (Korona-Glowniak et al., 2019).

In the current study, it was found that more virulent *H. pylori* strains (s1 and s1m1) and older patients(≥54) were strongly associated. This conclusion is in line with the findings of the reports from El-Shenawy et al. and Feliciano et al.. (El-Shenawyet al., 2017, Felicianoet al., 2015).

The bateria strains have been characterized as undergoing recombination with other more virulent and better adapted strains to host alterations, resulting in genotypes with a varied distribution throughout age groups (Karbalaeiet al., 2022, Husseinet al., 2010). Patients aged ≤ 37 and 38–53 had a statistically significant correlation with genotypes *s2* and *s2m2*, respectively. Our findings show that the *H. pylori vacA* genes differ in their sensitivity to clarithromycin. The results show that *s1m1* and *s1m2* mosaic combinations are less resistant to clarithromycin than s2m2 (75.9 %, p-value 0.0001) bacteria. It's possible that the presence of s2m2 types, which cause fewer inflammations in the host gastric epithelia, is the cause of the lower antibiotic delivery, which could make eliminating *H. pylori* more difficult. Our results were in line with those of Sugimoto and Yamaoka (Sugimoto and Yamaoka, 2009, Wanget al., 2017).

## 5 Conclusion

Finding *H. pylori* virulence markers may aid in identifying high-risk patients because *H. pylori* infection is prevalent among Iraqi patients with gastrointestinal illnesses. In the individuals under consideration, the most common vacA gene combinations were *s2m2* and *s1m1*, which were largely linked to gastritis and mixed gastric and duodenal ulcers. While the more pathogenic *s1* and *s1m1 H. pylori* strains were shown to have a significant connection with older patients aged ≥54 the less virulent *s2* and s2m2 *H. pylori* genotyping were detected in individuals aged ≤37 and 38–53. Clarithromycin resistance is higher in *s2m2* genotyped bacteria than in *s1m1* and *s1m2* mosaic combinations. It is essential for the effective eradication of the bacteria and the avoidance of serious effects like carcinoma of the stomach that *H. pylori* virulence variables and genes linked to drug resistance are characterized. This will lead to the development of particular medicines for *H. pylori* infections. Finally, the following were some of the limitations of our study: 1) vacA d and i alleles; 2) ignorance of cagA, dupA, and other bacterial virulence factors.

## Declaration of competing interest

The authors declare that they have no known competing financial interests or personal relationships that could have appeared to influence the work reported in this paper.
